# Measuring NQO1 Bioactivation Using [^2^H_7_]Glucose

**DOI:** 10.3390/cancers13164165

**Published:** 2021-08-19

**Authors:** Rohit Mahar, Mario C. Chang, Matthew E. Merritt

**Affiliations:** Department of Biochemistry and Molecular Biology, College of Medicine, University of Florida, Gainesville, FL 32610, USA; rmahar@ufl.edu (R.M.); marioc14@ufl.edu (M.C.C.)

**Keywords:** cancer, ^2^H-NMR, β-lapachone, [^2^H_7_]glucose, HDO, cell culture

## Abstract

**Simple Summary:**

Cancer is often characterized by profound changes in metabolism, some of which are appropriate targets for therapeutic intervention. Many cancers overexpress the two-electron reductase NQO1, which can bioactivate the drug β-lapachone, inducing a futile redox cycle that liberates large amounts of reactive oxygen species and results in subsequent cell death. However, β-lapachone has off-target toxicities in red blood cells, which makes minimal dosing for chemotherapeutic response desirable. Here, we show that magnetic resonance-based detection of [^2^H_7_]glucose metabolism provides a robust metric of NQO1 activation, as the redox perturbation causes downregulation of glycolytic flux that is detectable in the HDO and lactate signals. Imaging of either metabolic product could provide constraints for a continual reassessment model for controlling therapeutic dosing levels.

**Abstract:**

Treatment of cancers with β-lapachone causes NAD(P)H: quinone oxidoreductase 1 (NQO1) to generate an unstable hydroquinone that regenerates itself in a futile cycle while producing reactive oxygen species (ROS) in the form of superoxide and subsequently hydrogen peroxide. Rapid accumulation of ROS damages DNA, hyperactivates poly-ADP-ribose polymerase-I, causes massive depletion of NAD^+^/ATP, and hampers glycolysis. Cells overexpressing NQO1 subsequently die rapidly through an NAD^+^-keresis mechanism. Assessing changes in glycolytic rates caused by NQO1 bioactivation would provide a means of assessing treatment efficacy, potentially lowering the chemotherapeutic dosage, and reducing off-target toxicities. NQO1-mediated changes in glycolytic flux were readily detected in A549 (lung), MiaPaCa2 (pancreatic), and HCT-116 (colon) cancer cell lines by ^2^H-NMR after administration of [^2^H_7_]glucose. The deuterated metabolic products ^2^H-lactate and HDO were quantified, and linear relationships with glucose consumption for both products were observed. The higher concentration of HDO compared to ^2^H-lactate allows for more sensitive measurement of the glycolytic flux in cancer. Gas chromatography-mass spectrometry analysis agreed with the NMR results and confirmed downregulated energy metabolism in NQO1^+^ cells after β-lapachone treatment. The demonstrated method is ideal for measuring glycolytic rates, the effects of chemotherapeutics that target glycolysis, and has the potential for in vivo translation.

## 1. Introduction

Metabolic reprogramming is one of the key hallmarks of cancer cell metabolism [1,2]. Multiple cancers display an increased glucose uptake under normoxic conditions and favor lactate production as opposed to aerobic respiration (i.e., the Warburg effect) [3]. These metabolic defects have been studied extensively as a way to differentiate cancer cells from healthy cells, both for diagnosis and treatment purposes [4,5]. Among many therapeutic targets, NAD(P)H: quinone oxidoreductase 1 (NQO1) is an enzyme involved in cellular detoxifying reactions which has been recognized as a potential target for cancer treatment [6,7,8]. β-lapachone is a bioactivatable chemotherapeutic agent that directly targets cancer cell lines over-expressing NQO1, leading to the interruption of glycolytic metabolism and changes in the redox state of the cell [9]. NQO1 quinone detoxification is highly dependent on the stability of the hydroquinone generated from the two-electron reduction it catalyzes [10]. β-lapachone treatment takes advantage of the NQO1 catalyzed reaction through the formation of an unstable hydroquinone that regenerates itself while producing peroxides in a futile cycle [11]. This process causes the rapid accumulation of reactive oxygen species (ROS), leading to DNA damage, followed by hyperactivation of poly(ADP-ribose) polymerase-1 (PARP1), leading to NAD^+^ and ATP loss, and cell death by a mechanism known as NAD^+^-keresis [12]. β-lapachone-induced cytotoxicity directly involves the inhibition of the NAD^+^-dependent glyceraldehyde 3-phosphate dehydrogenase (GAPDH), which suppresses overall glycolytic flux [6,12]. While β-lapachone has shown significant promise in Phase-1 clinical trials, off-target, dose-dependent toxicity in red blood cells due to methemoglobinemia has caused significant safety concerns [13]. The drug β-lapachone causes a profound interruption of glycolytic metabolism, which makes this pathway a prime target for metabolic imaging. A means of assessing NQO1 bioactivation in vivo could empower adaptive dosing strategies [14] that could reduce the amount of agent needed while maintaining its anti-cancer benefits. The glucose analog [^18^F]2-fluoro-2-deoxy-glucose (^18^FDG) is widely used clinically with positron emission tomography (FDG-PET) to produce images of glucose uptake in tumors, but it does not report on downstream metabolism, and causes radioactive exposure that carries a risk for secondary cancer [15,16]. Without the ability to monitor not only glucose uptake, but also its downstream metabolism, FDG-PET is an unsuitable avenue for imaging NQO1 activation. Recently, we showed that HDO production from [^2^H_7_]glucose could be utilized as a marker to quantitatively identify cancer metabolism [17]. We utilized hepatocellular carcinoma HUH-7 cells and normal AML-12 hepatocytes, and showed that HDO production significantly increased in HUH-7 cells compared to normal AML-12 cells [17]. While lactate production has been used as an indicator of upregulated glycolysis, the previous study showed HDO is a sensitive marker that can be applied as a safer alternative to radiotracer techniques [17]. We also demonstrated this approach is suitable for brain imaging in vivo in a rat model [18]. With the recent demonstration of ^2^H imaging as an effective means for detecting cancer in vivo in humans [19], extension of this method to detect the action of chemotherapeutics is logical.

Here, we utilized A549 lung carcinoma and MiaPaCa2 pancreatic cancer cells, which express high levels of NQO1, and HCT-116 colon cancer cells with low levels of NQO1, to test the hypothesis that [^2^H_7_]glucose could be used as a metabolic contrast agent sensitive to a β-lapachone-mediated reduction in glycolytic flux. NQO1 expression levels in these cancer cells were established previously by Western blot analysis [11,20], and shown to be sustained in cancer cells after β-lapachone treatment [9,21]. Additionally, we confirmed that these cell lines do not express the C609T polymorphism, which affects NQO1 activity [22,23,24]. Experimentally, we quantitatively measured decreased glycolytic rates in β-lapachone treated cancer cells using HDO, ^2^H-lactate, and residual [^2^H_7_]glucose in cell culture media. The estimates of HDO production rate combined with the production of the ^2^H-lactate results in a comprehensive accounting of the [^2^H_7_]glucose metabolism in control and β-lapachone treated cells. HDO production is linear with lactate production, but is characterized by a much larger change in absolute signal intensity, i.e., greater dynamic range. Our previous work demonstrated this method in normal versus cancerous cells. Here, HDO detection is extended as a method for monitoring chemotherapeutic inhibition of glycolysis.

## 2. Materials and Methods

### 2.1. Cell Lines, Chemicals, and Media

A549 (lung), MiaPaCa2 (pancreatic), and HCT-116 (colon) cancer cells were purchased from the American Type Culture Collection (ATCC, Manassas, VA, USA). Dulbecco’s Modified Eagle Medium (DMEM), Phosphate Buffered Saline (PBS) and Dimethyl Sulfoxide (DMSO) were purchased from Thermo Scientific (Waltham, MA, USA). Fetal Bovine Serum (FBS) was purchased from Atlas Biological (Fort Collins, CO, USA). Deuterated pyrazine (Pyrazine-D_4_), D-glucose, [1,2,3,4,5,6,6-^2^H_7_]-D-glucose ([^2^H_7_]glucose), and 2,2-dimethyl-3,4-dihydrobenzo[h]chromene-5,6-dione (β-lapachone) were purchased from Sigma Aldrich, (St. Louis, MO, USA). Methoxyamine hydrochloride in pyridine (MOX reagent) and N-methyl-N-(tert-butyldimethylsilyl)-trifluoroacetamide + 1% tertbutyldimethylchlorosilane (MTBSTFA + TBDMS) were purchased from Thermo Scientific, (Waltham, MA, USA).

### 2.2. Cell Culture

A549, MiaPaCa2, and HCT-116 cell lines were maintained in a complete growth medium composed of Dulbecco’s Modified Eagle Medium (DMEM) with 10% *v/v* FBS, 50 µg/mL penicillin, 50 µg/mL streptomycin, 10 µg/mL neomycin. Cell lines were cultured at 37 °C in a 95% air and 5% CO_2_ atmosphere in an air-jacketed incubator (Heracell Vios 160i, Thermo Scientific, Waltham, MA, USA). Every 3 days growth media was replenished, and once at 80% confluence cells were subcultured 1:10 into six 100 mm OD (56.7 cm^2^ culture area) cell culture plates. All cell lines were grown to 70–80% confluency (~10 million total cells) and washed once with warm PBS and incubated with 4 mL each of either DMEM with 5.5 mM [^2^H_7_]glucose and 6 µM β-lapachone (dissolved in DMSO) or DMEM with 5.5 mM [^2^H_7_]glucose and DMSO vehicle for 2 h. During the 2 h treatment, 100 μL aliquots were collected at 0 min, 15 min, 60 min, and 2 h. After the 2 h time point, all DMEM was collected and cells were washed once with warm PBS and incubated with 4 mL each of DMEM with 5.5 mM [^2^H_7_]glucose for 6 h, withdrawing 100 µL aliquots at 0 min, 30 min, 2 h, and 6 h. After the 6 h time point cells were trypsinized and collected for O_2_ consumption measurements (Supplementary Figure S8). An Oxygraph^+^ System from Hansatech Instruments (Pentley, KL, UK) was used to measure O_2_ consumption by analyzing 1.5 mL of the harvested cell suspension in a 37 °C sealed water chamber connected to an S_1_ Oxygen Electrode Disc that measured voltage differences in the suspension based on the amount of O_2_ present in solution.

### 2.3. NMR Sample Preparation

Cell media samples were prepared without an extraction. Each cell media sample of A549, MiaPaCa2, and HCT-116 cells was spiked with a 25 mM pyrazine-D_4_ internal standard stock solution to attain a final concentration of 2.5 mM. Addition of pyrazine-D_4_ internal standard allowed for quantification of HDO, ^2^H-lactate, and unconsumed [^2^H_7_]glucose from ^2^H-NMR data. A total of 50 μL of cell media sample for each time point was transferred into 1.7 mm NMR sample tubes for NMR data acquisition.

### 2.4. ^2^H-NMR Spectroscopy

A Bruker 14.1 T magnet system equipped with an Avance Neo Console (Bruker Biospin) and 1.7 mm CryoProbe was used to acquire ^2^H-NMR data of the deuterium-labeled metabolites in the cell media samples. The deuterium lock channel was used to acquire the ^2^H-NMR spectra at 92.12 MHz resonant frequency. An acquisition time (AQ) of 1 s and a relaxation delay (d1) of 2 s (total, 3 s of repetition time) with a 90° pulse was used to acquire all ^2^H-NMR spectra. A total of 4096 complex data points were digitized with the spectral width of 11 ppm using 256 scans for each of the 12 FIDs (3072 scans) for each of the samples. All NMR data were collected at room temperature (25 °C).

### 2.5. ^2^H-NMR Data Processing and Quantification of HDO, ^2^H-Lactate, and Residual [^2^H_7_]Glucose

TopSpin 4.0.3 was used for data acquisition. ^2^H-NMR spectra were transferred to MestReNova *v*14.0.1-23284 (Mestrelab Research S.L.) for further processing. The ^2^H-NMR spectra were refined by adjusting the exponential window function to 0.5 Hz and increasing the zero-filling of the free induction decay (FID) to 8192 data points before the Fourier Transform (FT). Manual phase corrections were made for each spectrum, as well as automatic spline baseline corrections. A total of 12 FIDs were acquired for each sample and all 12 ^2^H-NMR spectra were aligned with respect to the pyrazine-D_4_ peak and combined to account for peak shifting due to magnetic field drift over the course of the experiment. Concentrations of HDO, ^2^H-lactate and residual [^2^H_7_]glucose in the cell media were calculated using peak areas of the internal standard pyrazine-D_4_. The concentration of pyrazine-D_4_ (2.5 mM) was used to quantify the concentration of HDO and residual [^2^H_7_]glucose, normalized to the number of deuterons responsible for corresponding resonances. ^2^H-lactate concentration was corrected for mono- and di-deuterated lactate isotopomers using the fractional enrichments of lactate m+1 and m+2 mass isotopologues. The time-series data were imported into GraphPad Prism and linear regression statistical analysis was performed. Simultaneously, the significant difference between the slopes of control and treatment groups was determined. All linear regression analysis was performed in Graphpad Prism (GraphPad Software, La Jolla California USA, www.graphpad.com).

### 2.6. GC-MS Sample Preparation

Media (20 µL) samples were dried in the Reacti-vial reaction vials under nitrogen gas airflow. Cells were extracted with 1 mL of Acetonitrile:Isopropanol:Water (3:3:2, *v*:*v*:*v*), then centrifuged for 15 min at 10,000× *g* at 4 °C. The supernatant was transferred into a new centrifuge tube and dried in a speedvac. Dried cell extract was resuspended in 0.5 mL of Acetonitrile:Water (1:1, *v*:*v*) and centrifuged for 5 min at 10,000× *g*. The supernatant was transferred into Reacti-vial reaction vials and dried down under nitrogen gas airflow. DL-Norleucine was added as an internal standard. Fifty microliters of 2% MOX reagent were added to the vial and vortex for 5 s followed by incubation at 30 °C for 90 min on a heating block with a magnetic stirrer for faster reaction and smooth mixing of the samples. Fifty microliters of MTBSTFA +TBDMS were added to each vial and incubated at 60 °C for 1 h. Reacti-vials were centrifuged at 8000× *g* for 10 min and the supernatant was transferred into GC vials.

### 2.7. GC-MS Analysis

GC-MS analysis was performed with Thermo Scientific ISQ Single Quadrupole Mass Spectrometer and Trace 1310 Gas Chromatograph (GC-MS, Thermo Scientific, USA) equipped with 30 m long Restek 95% dimethyl/5% diphenyl polysiloxane RTX-5MS column, 0.25 mm internal diameter, 0.25 µm film, with 10 m empty guard column (Restek, Bellefonte, PA, USA). The initial oven temperature was 60 °C for 60 s and the ramp oven temperature increased to 325 °C at 10 °C/min with 5 min final hold time at the end. Ion source temperature was maintained at 230 °C with electron ionization energy at 70 eV and a quadrupole mass analyzer was utilized for separation of ions. Helium was used as carrier gas at a flow rate of 1 mL/min. The Mass Isotopomer Distribution (MID) of the lactate and citrate was obtained by integration of extracted ion chromatograms from each of the isotopologues. The MID of lactate and citrate were corrected for natural isotope abundance using the Isotopomer Network Compartmental Analysis (INCA) software [25]. Extracellular and intracellular metabolite’s total intensities were normalized to the intensity of DL-Norleucine internal standard and compared between control and treated groups. Significant differences for metabolites between control and treatment groups were established using Student’s t-test and FDR correction utilizing RStudio (RStudio Version 1.4.1717, PBC, Boston Massachusetts, USA, www.rstudio.com (accessed on 17 August 2021)).

## 3. Results

### 3.1. ^2^H-NMR Spectra of Cell Culture Media

**Figure 1:**
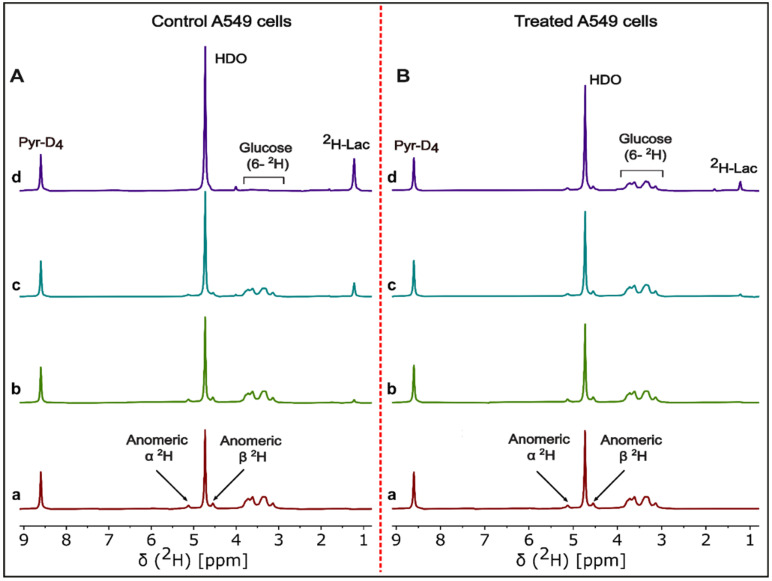
Representative ^2^H-NMR spectra of cell media time course analysis. Stacked plot of ^2^H-NMR spectra of DMSO control (left, panel **A**) and β-lapachone treated (right, panel **B**) of the A549 cell line incubated with 5.5 mM [^2^H_7_]glucose. Cell media samples were withdrawn at (**a**) 0 min, (**b**) 30 min, (**c**) 2 h, and (**d**) 6 h incubation time points of the β-lapachone post-treatment cultures of A549 cancer cells. Labeling in the (a and d) spectra of panel **A**,**B** shows the signals for pyrazine-D_4_, HDO, residual [^2^H_7_]glucose, and ^2^H-lactate.

^2^H-NMR spectra were recorded as a function of time for cell media samples during treatment and post-treatment periods for A549, MiaPaCa2 and HCT-116 cancer cell lines. HDO and ^2^H-lactate signal intensity increase with incubation time while [^2^H_7_]glucose signal decreases for DMSO control A549 cells (Figure 1A). Signal intensities of HDO and ^2^H-lactate increase slowly with incubation time, which matches a slow decrease of [^2^H_7_]glucose signal intensity in β-lapachone treated A549 cells (Figure 1B).

### 3.2. Quantification of the ^2^H-Lactate, HDO and [^2^H_7_]Glucose in Cell Media

**Figure 2:**
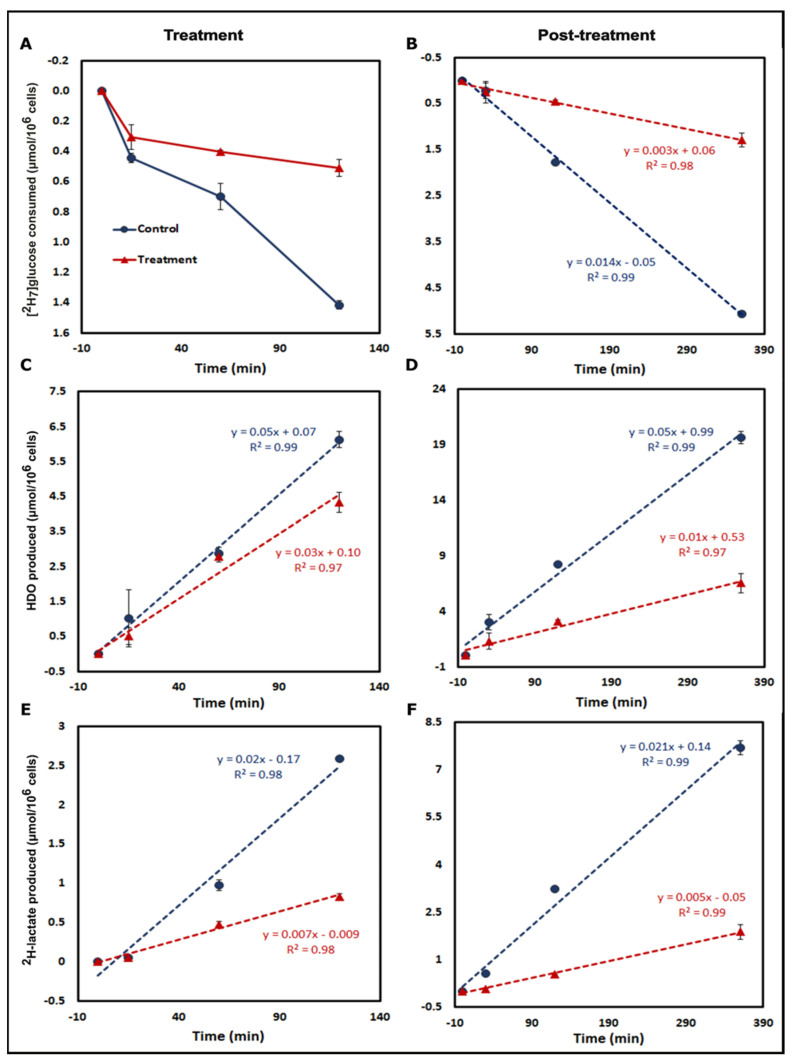
Time course analysis of [^2^H_7_]glucose metabolism. Kinetic plots of treatment (1st column) and post-treatment (2nd column) [^2^H_7_]glucose metabolism by control and treated A549 cells incubated in DMEM with 5.5 mM [^2^H_7_]glucose. Analysis of [^2^H_7_]glucose consumption for both periods is shown in (**A**,**B**). The kinetics of HDO production are displayed in panels (**C**,**D**). Similarly, kinetic data for ^2^H-lactate production are shown in panels (**E**,**F**). Results were calculated from ^2^H-NMR data of each of the cell media samples including a blank withdrawn at the 0 min time point for each time period. (Note: [^2^H_7_]glucose consumption and HDO and ^2^H-lactate production were measured in the cell media samples withdrawn at 0 min, 15 min, 1 h, and 2 h for treatment and 0 min, 30 min, 2 h, and 6 h time points of the post-treatment period. Three biological replicates were used for each group and data are represented as mean ± standard error of mean (SEM)).

**Figure 3:**
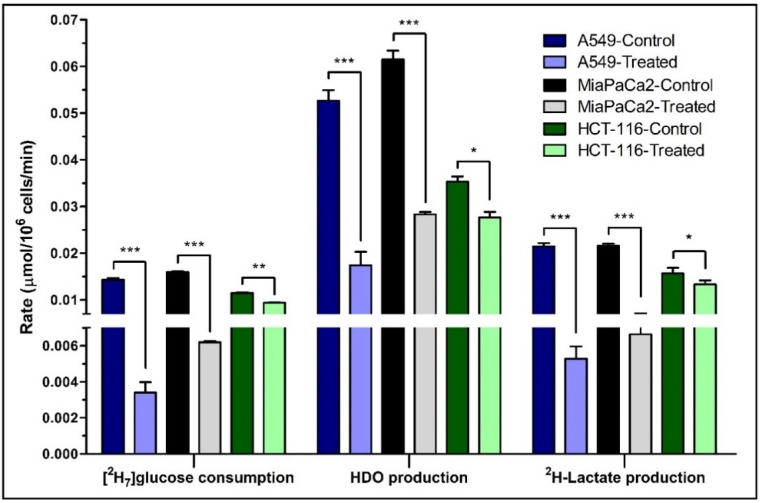
The rates of [^2^H_7_]glucose consumption and HDO/^2^H-lactate production. The consumption rate of [^2^H_7_]glucose and production rates of HDO and ^2^H-lactate are shown for the post-treatment period. Rates were calculated from the kinetic data of control and β-lapachone treated A549, MiaPaCa2 and HCT-116 cancer cells. Biological triplicate data are represented as mean ± SEM. Significance levels between the slopes of control and treatment groups were determined using the statistical analysis in Graphpad Prism and shown as: “*” if *p* ≤ 0.05, “**” if *p* ≤ 0.01, and “***” if *p* ≤ 0.001.

[^2^H_7_]glucose consumption was significantly higher in control cells at the 120 min time point for both treatment and post-treatment periods (Figure 2 and Figure S1A,B, Tables S1 and S2). For the post-treatment period, [^2^H_7_]glucose consumption rates for control and treated A549 cells were 0.014 ± 0.0004 and 0.003 ± 0.0006 μmol/10^6^ cells/min (Figure 2B and Figure 3), respectively, with a significantly higher rate in control cells (*p* = 0.0001). Linearity for ^2^H-lactate and HDO production from A549 cells into the culture medium is excellent with an R^2^ of 0.99. HDO production was corrected for the natural abundance concentration in each cell media sample. The production rates of HDO for A549 cells were 0.050 ± 0.005 and 0.037 ± 0.002 μmol/10^6^ cells/min for control and treated cells, respectively, and were significant (*p* = 0.02) between both groups in the treatment period (Figure 2C). In the post-treatment period, HDO production rates were significantly higher in A549 control cells (0.053 ± 0.002 and 0.017 ± 0.003 μmol/10^6^ cells/min, respectively, *p* = 0.0001) (Figure 2D). The production rates of ^2^H-lactate in control and treated cells during treatment were also significantly different (0.02 ± 0.0005 and 0.007 ± 0.004 μmol/10^6^ cells/min, respectively, *p* = 0.0001) (Figure 2E and Figure 3). The post-treatment period rates were 0.02 ± 0.001 and 0.005 ± 0.001 μmol/10^6^ cells/min, respectively, (*p* = 0.0001) (Figure 2F). Similar results were shown in the MiaPaCa2 cells (Figure 3, Tables S1 and S2).

HCT-116 cells express less NQO1, and we hypothesized lower significant differences between control and treated cells for glucose consumption and production of HDO and ^2^H-lactate after β-lapachone exposure (Figure 3 and Table S1). Despite lower levels of NQO1, HCT-116 cells still showed significant differences on treatment, though less robustly than the A549 and MiaPaca2 cell lines. In general, higher NQO1 expression [11,19] correlates with greater differences in glycolytic rates between control versus treatment as expected in A549 and MiaPaCa2 cell lines (Figure 3, Tables S1 and S2).

### 3.3. Correlation between [^2^H_7_]Glucose Consumption and ^2^H-Lactate, HDO Production

**Figure 4:**
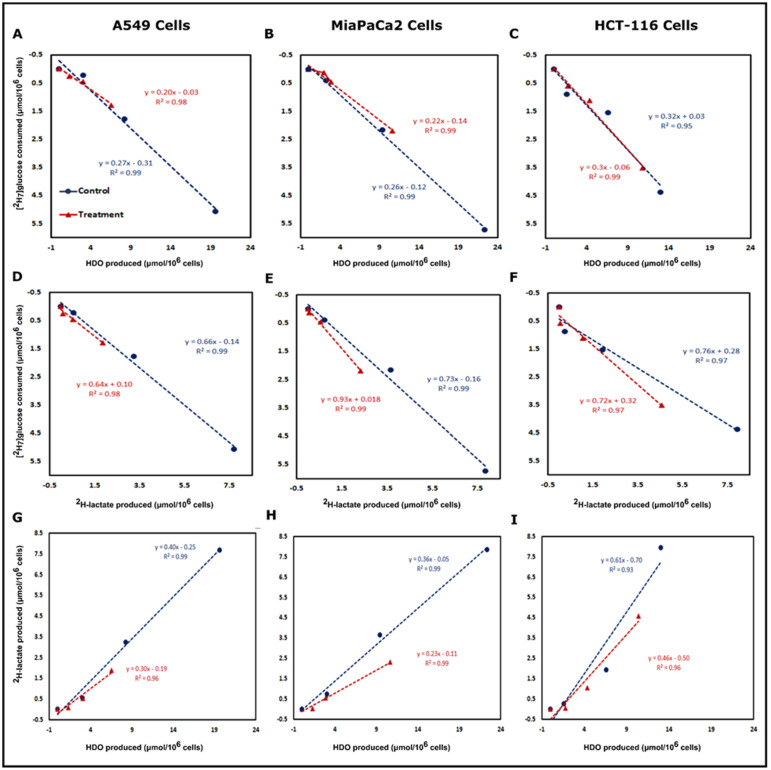
HDO and ^2^H-lactate production are directly related to [^2^H_7_]glucose consumption. Correlation plots of [^2^H_7_]glucose consumption vs. HDO production (top row) and ^2^H-lactate production (middle row) and correlation between ^2^H-lactate vs. HDO production (bottom row) are shown for the post-treatment period of A549 (Panels **A**,**D** and **G**), MiaPaCa2 (Panels **B**,**E** and **H**) and HCT-116 (Panels **C**,**F** and **I**). Correlations in all conditions were linear with R^2^ values > 0.96. Correlation plots were plotted from the calculated concentration of consumed [^2^H_7_]glucose and produced ^2^H-lactate and HDO by control and treated cells. The excellent linearity between ^2^H-lactate and HDO production and higher production of HDO demonstrates that the HDO could be a much more sensitive marker than lactate for monitoring NQO1 bioactivation.

Correlation plots were made for the post-treatment period to determine relationships between glucose consumption and the production of the downstream metabolites. In Figure 4, panel A and D clearly show the linearity between [^2^H_7_]glucose consumption and HDO or ^2^H-lactate production for A549 cells, respectively. This behavior is preserved even in β-lapachone treated cells. The concentration of produced HDO was higher than ^2^H-lactate, producing a metric of glycolysis with a greater dynamic range than lactate (Figure S1 and Table S2). The sensitivity of HDO production over ^2^H-lactate production with respect to the [^2^H_7_]glucose consumption is also evidenced by the slopes in Figure 4. In HCT-116 cells, 0.32 [^2^H_7_]glucose units are required to produce an HDO and 0.76 to produce lactate. Similar trends were found in the case of A549 and MiaPaCa2 cells (Figure 4). Additionally, the linear trends between [^2^H_7_]glucose consumption and HDO/^2^H-lactate production led us to calculate the ^2^H mass balance of consumed [^2^H_7_]glucose and its incorporation into ^2^H-lactate and HDO at the 6 h time point. In the case of A549 cells, mass balance accountability is lower (84.1%) than treated cells (92.4%) whereas, in the case of HCT-116 cells, where NQO1 is less abundant, it was 68.4 and 70.8% for control and treated cells, respectively (Table 1).

### 3.4. Correlation between ^2^H-Lactate and HDO Production

Correlation plots between HDO and ^2^H-lactate production for all three cancer cell lines demonstrated linearity between control and treated cells in the post-treatment phase (Figure 4). In A549 cells, 0.40 ^2^H-lactate was produced per HDO, which drops to 0.30 lactate/HDO in the β-lapachone treatment group. However, HDO concentration is more than 2× than that of the ^2^H-lactate (Figure 4G). Similarly, MiaPaCa2 and HCT-116 cells also demonstrated a linear correlation between HDO and ^2^H-lactate production in the control and treated cells post-treatment (Figure 4H,I). The linear correlation between lactate and HDO production was excellent with R^2^ of 0.99 for control A549 and MiaPaCa2 cells, whereas treated A549 and MiaPaCa2 cells showed linearity with R^2^ of 0.96 and 0.99, respectively (Figure 4G,H). The linear correlation was slightly lower for HCT-116 cells with R^2^ of 0.93 and 0.96 for control and treated cells, respectively (Figure 4I).

### 3.5. Fractional Enrichment of Lactate and Citrate Isotopologues

**Figure 5:**
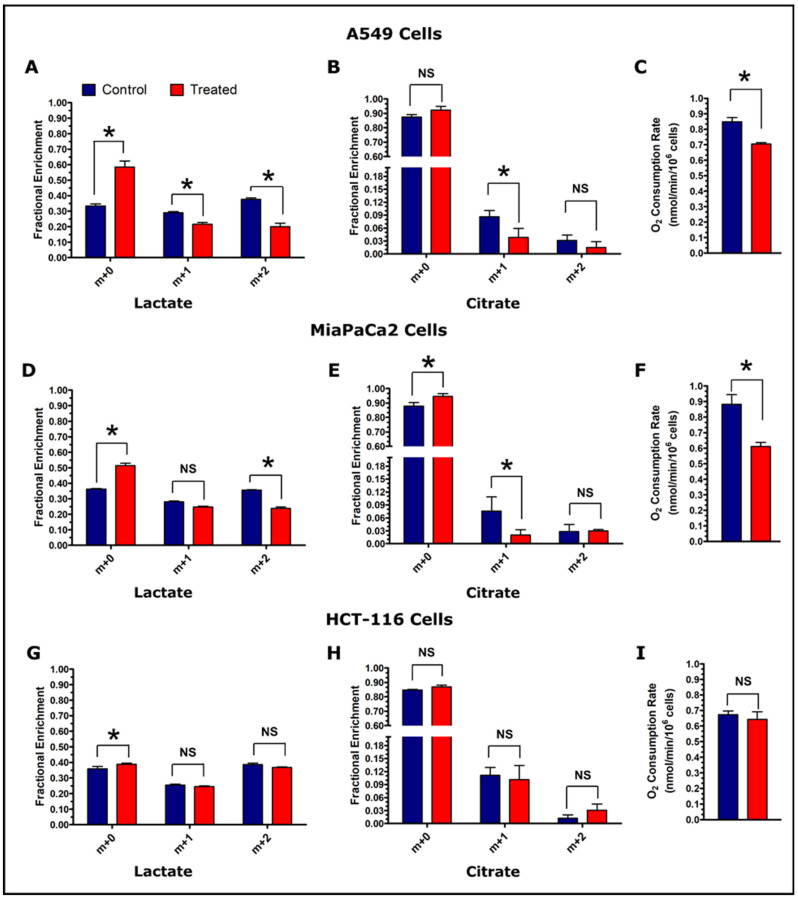
Assessment of glycolytic and TCA cycle activity in control and treated cells. Fractional enrichment of lactate and citrate, and O_2_ consumption rate are shown for A549 cells (Panels, **A**–**C**), MiaPaCa2 cells (Panels, **D**–**F**), and HCT-116 cells (Panels, **G**–**I**). Enrichment results were calculated from the GC-MS data of cell media samples collected at the 360 min post-treatment time point. The GC-MS data were corrected for natural isotope abundance using the Isotopomer Network Compartmental Analysis (INCA) software. (Note: *N* = 3: biological replicate data are represented as mean ± SEM. Significant levels were calculated using the Student’s t-test between control and treated groups and are displayed as: non-significant “NS” if *p* > 0.05 and “*” if *p* ≤ 0.05. Oxygen consumption was measured at the end of tracer experiment (total time period of 8 h) and O_2_ consumption rates were calculated for the number of cells used for the measurements and normalized per million cells).

Natural isotope abundance corrected fractional enrichments of lactate and citrate isotopologues at 360 min time points were analyzed for assessing glycolytic and TCA cycle activity. A549 cells showed significant differences in all lactate isotopologue patterns (Figure 5A). The citrate m+1 isotopologues were significantly higher in control A549 cells (Figure 5B). Lactate m+2 and citrate m+1 isotopologues were significantly higher in control MiaPaCa2 cells (Figure 5D,E). HCT-116 cells showed insignificant differences for lactate and citrate (m+1 and m+2) isotopologues whereas lactate m+0 isotopologues were significantly lower in control HCT-116 cells (Figure 5G,H).

### 3.6. Oxygen Consumption Rate of the Cancer Cell Lines

Oxygen consumption is a general index of metabolic turnover, and was measured after the completion of the tracer experiment. The O_2_ consumption rates of A549 and MiaPaCa2 control cells were found significantly higher in treatment cells (Figure 5C,F). On the other hand, HCT-116 cells did not show a significant difference in O_2_ consumption at the conclusion of the experiment (Figure 5I).

### 3.7. Metabolic Profiling of Control and β-Lapachone Treated Cancer Cell Lines

**Figure 6:**
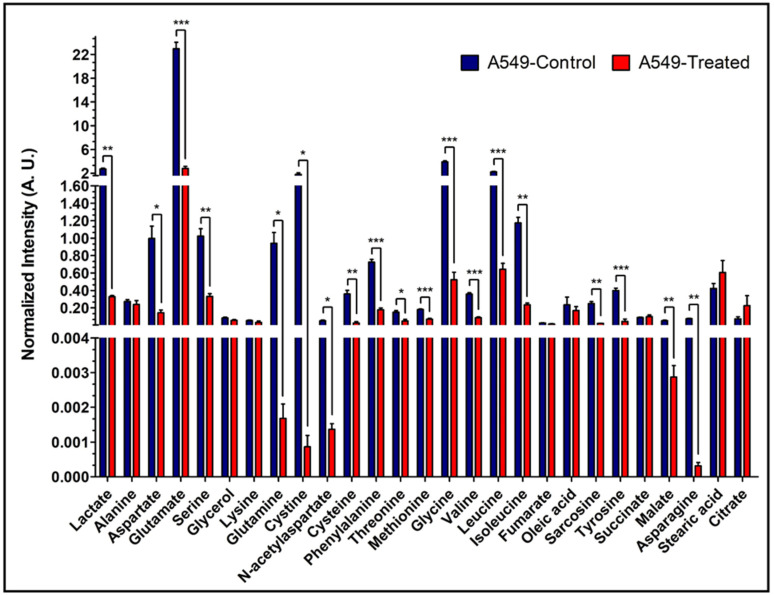
Intracellular levels of the metabolites in A549 cells. Bar plots of GC-MS analysis-based levels of different intracellular metabolites from A549 cells. Shown are the normalized intensities of the differential levels of metabolites between control and treated A549 cells. (Note: *N* = 3: biological replicate data are represented as mean ± SEM. Student’s t-test was used to calculate statistical significance followed by false discovery rate (FDR) performed in RStudio, to adjust *p* values and represented as: “*” if *p* ≤ 0.05, “**” if *p* ≤ 0.01, and “***” if *p* ≤ 0.001).

To assess the consumption and production of extracellular metabolites, 6 h cell media samples were analyzed by GC-MS. Eighteen key metabolites were identified and compared between control and treatment groups across all three cancer cell lines. More extracellular metabolites were found to be significantly different among treatment and control groups for NQO1^+^ cells, A549 and MiaPaCa2 (Figures S4 and S5), than for NQO1^-^ HCT-116 cells (Figure S6). Notably, lactate levels were significantly lower in treatment group media for both A549 (*p* = 0.03) and MiaPaCa2 (*p* = 0.01) but showed the opposite non-significant trend in HCT-116 cell media (Figures S4–S6). Analysis of cell media showed higher levels of glycine, tyrosine, glutamine, serine, isoleucine, leucine, and valine in treated cells. To assess intracellular metabolite levels, GC-MS analysis was conducted on A549 and HCT-116 cell extracts. More metabolites with greater significant differences were detected between the control and treatment groups of A549 cells compared to HCT-116 cells. The intracellular levels of lactate agree with the analysis of lactate production shown in Figure 3. Treated A549 cells contained ~10 times less lactate than control cells (*p* = 0.003) (Figure 6), whereas treated HCT-116 cells contained ~1.75 times less lactate than control cells (*p* = 0.004) (Figure S7). Treated A549 cells also showed significantly lower levels of some TCA cycle intermediates, polar amino acids, and branched-chain amino acids (Figure 6). Both A549 and HCT-116 cells showed significantly lower levels of glutamine in treated cells (Figure 6 and Figure S7). Most of the intracellular metabolites showed insignificant differences between control and treated HCT-116 cells.

## 4. Discussion

Deuterium NMR was used sporadically in the late 1980s, [26,27] but has re-emerged as a method for measuring turnover in highly metabolic systems. Recently, deuterium (^2^H) magnetic resonance imaging (DMI) was used to assess cancer metabolism, as it provides requisite chemical and temporal resolution [19,28]. The low natural abundance (0.0115%) of deuterium atoms [29] maximizes the specificity of deuterated tracers for metabolic flux measurements in cell culture and in vivo studies. Deuterium is a non-radioactive nucleus, making perdeuterated glucose (or [^2^H_7_]glucose) a safe tracer for *in vitro* and in vivo studies. It has been demonstrated that the total glycolytic rate is not affected by perdeuteration in glucose [30], suggesting negligible effects on the total glycolytic rate in these experiments. With this experimental design, we sought to determine if [^2^H_7_]glucose could be used to measure changes in glycolysis after administration of β-lapachone, which is known to deplete NAD^+^ stores in NQO1^+^ cells [11]. While β-lapachone has demonstrated powerful effects in a variety of cancer types, it also has known off-target effects on the red blood cell [13]. The common side-effect of hemolytic anemia limits drug dosing and has caused some clinical trials to be terminated early. The adaptive dose-finding approach to maximize efficacy while minimizing toxicities can be significantly amplified with stronger parametrization of favorable results [31]. Because of the strong, logical connection between β-lapachone action and decreased glycolytic flux, the analytical methods we demonstrate here suggest themselves as potential biomarkers for input into an adaptive dosing paradigm. Using this specific model, we demonstrate a global accounting of perturbations in glycolysis by measuring HDO and lactate production. This method produces a more satisfactory accounting of glucose metabolism than observing lactate alone, which in the case of β-lapachone action is doubly inhibited by the loss of the NAD^+^ cofactor at GAPDH and LDH.

The NMR data (Figure 1) have a high signal-to-noise ratio, allowing very exact estimates of concentration. Time series data showed a quantitative estimate of increased glucose consumption in control cancer cells based on increased HDO and ^2^H-lactate and decreased residual [^2^H_7_]glucose signals. Figure 1B showed reduced signal intensities for HDO and ^2^H-lactate compared to control cells (Figure 1A), indicating the effects of NAD^+^ depletion on glycolytic flux [6,11].

**Figure 7:**
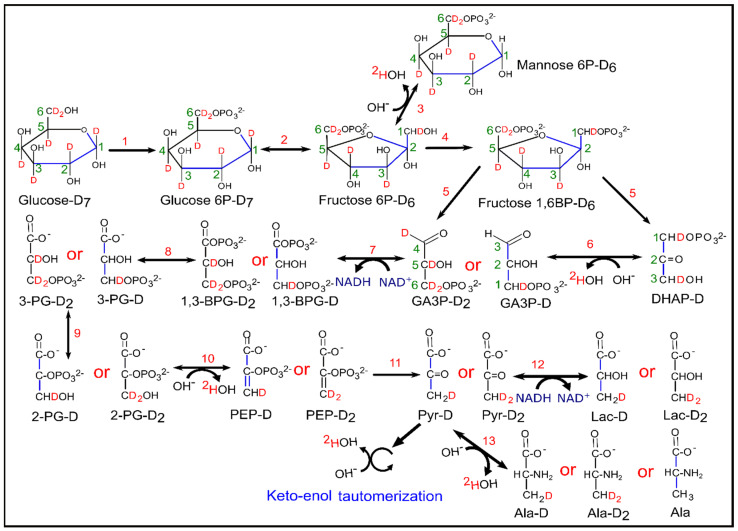
Schematic diagram of glycolysis and evolution of HDO from [^2^H_7_]glucose. Enzymatic reactions indicated by numbers in the figure were catalyzed by (1) hexokinase, (2) phosphoglucose isomerase, (3) phosphomannose isomerase, (4) phosphofructokinase, (5) aldolase, (6) triose phosphate isomerase, (7) glyceraldehyde 3-phosphate dehydrogenase, (8) phosphoglycerate kinase, (9) phosphoglyceromutase, (10) enolase, (11) pyruvate kinase, (12) lactate dehydrogenase and (13) alanine amino transferase. (Note: number of deuterons, which ends up in the methyl group of lactate isotopomers were written with the name of lactate precursors from GA3P-D and GA3P-D_2_ onwards).

Apart from ^2^H-lactate, HDO also acts as a sensitive marker for glycolytic rate because the loss of ^2^H from [^2^H_7_]glucose occurs in many enzymatic processes during glycolysis, and therefore may provide a more global view of glucose utilization. Loss of ^2^H atoms from C6 of [6,6-^2^H_2_]glucose is mainly due to the catalytic action of pyruvate kinase (PK) whereas loss of ^2^H from C1 of [1-^2^H]glucose is due to both pyruvate kinase (PK) and phosphomannose isomerase (PMI) [32]. The presence of a ^2^H kinetic isotope effect at triose phosphate isomerase (TPI), results in more ^2^H liberation from dihydroxyacetone phosphate (DHAP) [33,34]. HDO is produced by TPI from the C2 position of GA3P and DHAP, within the course of direct metabolism of one equivalent of [^2^H_7_]glucose to pyruvate [17]. HDO production from the C1 and C2 positions can occur through the interconversion of fructose-6-phosphate (F6P) and mannose-6-phosphate (M6P) via PMI [35]. Enolase enzymatic activity on phospho-enol pyruvate (PEP) and keto-enol tautomerization in pyruvate are also prominent sites of HDO production during glycolysis [19,36,37,38,39,40]. Additionally, deuterium atoms could be lost during the conversion of pyruvate into alanine and lactate via alanine aminotransferase (ALT) and lactate dehydrogenase (LDH) reactions, respectively [41]. Figure 7 summarizes the reactions that generate ^2^H-lactate and HDO from [^2^H_7_]glucose during glycolysis. Apart from glycolysis, deuterium could also be lost to solvent water in the form of HDO in several reactions in the tricarboxylic acid (TCA) cycle [28,42].

Treatment of NQO1^+^ A549 cells with β-lapachone showed clear differences in [^2^H_7_]glucose consumption, ^2^H-lactate and HDO production between control and treatment groups (Figure 2). Both during treatment and post-treatment, HDO and ^2^H-lactate production is linear. NQO1/β-lapachone induced futile cycling consumes NADPH while producing large amounts of hydrogen peroxide that attack DNA and overwhelm repair mechanisms causing the hyperactivation of poly-(ribose-AMP) polymerase (PARP1), leading to a significant loss NAD^+^/ATP pools [20,24]. Thus, glycolytic enzymes that depend on NAD^+^/NADH like GAPDH and LDH are highly affected by β-lapachone treatment, leading to a reduction in glycolytic rate [11]. In NQO1^+^ cells, we observed that ^2^H-lactate production is drastically reduced. LDH catalytic dependence on NAD^+^/NADH suggests that the production of ^2^H-lactate does not reflect glucose utilization because it is doubly reduced by β-lapachone-dependent NAD^+^/NADH depletion; once by upstream GAPDH inhibition and secondly by an insufficient pool of NAD^+^/NADH cofactor for LDH. On the other hand, HDO serves as a better indicator of overall drug-dependent disruptions of glycolysis because multiple glycolytic enzymes are responsible for HDO production. Additionally, measurement of HDO production can denote distinguishable differences in drug-treated and non-treated cells with robust signals corresponding to high concentrations of HDO detected in extracellular media, exemplified in A549 and MiaPaCa2 cells (Figure 2 and Figure S2). The drug-dependent effects on glycolytic rate by β-lapachone treatment are verified by the response of NQO1^-^ HCT-116 cells showing lower differences in [^2^H_7_]glucose consumption and ^2^H-lactate/HDO production between treatment and control cells (Figure S3).

Correlation plots demonstrated an excellent linear slope for HDO production for both control and treatment cells, with a much higher concentration of HDO compared to ^2^H-lactate. The linear slopes show that much less [^2^H_7_]glucose is required to show the differences in glycolytic rates between control and treated cells using HDO as a marker (Figure 4). This reinforces the idea that HDO production can serve as a biomarker of β-lapachone action. Congruently, we observed a significant reduction in ^2^H-lactate production, which coincides with the implications of the direct effects of NAD^+^/NADH depletion on LDH. Theoretically, NADPH depletion might increase the flux of glucose-derived carbon through the pentose phosphate pathway (PPP). Previously, we showed the possible fate of [^2^H_7_]glucose for HDO/^2^H-lactate production, and the loss of ^2^H atoms from glycolysis, sugar isomerization, and potentially the PPP [17]. A recent publication using flux balance analysis demonstrated that the malic enzyme and isocitrate dehydrogenase are the most likely sources of NADPH that would be involved with the β-lapachone-mediated futile cycle [43]. Therefore, we do not expect the PPP to be a major source of HDO in our experimental conditions. The mass balance between [^2^H_7_]glucose consumption and HDO/^2^H-lactate production is close to 85–90%, so it is conceivable that some of the ^2^H-label is lost in other metabolic pathways. For example, ^2^H-labeling in metabolites involved in multiple pathways downstream of glucose is shown in Supplementary Figure S9. As these metabolites were not quantitated by standard addition, we cannot include them in the mass balance analysis.

The excellent linearity between ^2^H-lactate and HDO production shown in the correlation plots of Figure 4 demonstrate that glycolytic rate can be measured by HDO with high sensitivity even in the presence of interrupted metabolism—in this case, the loss of the NAD^+^ cofactor for LDH and GAPDH. Mass balance calculations were in good agreement for the more glycolytic A549 and MiaPaCa2 cell lines, but a significant undercounting of deuterium-labeled metabolites in the HCT-116 cells suggest that other pathways than glycolysis may be more active. Mass isotopologue analysis further confirmed reduced glycolysis and tricarboxylic acid (TCA) cycle metabolism in β-lapachone treated cells, as indicated by significantly lower labeling of deuterium in lactate and citrate isotopologues than control cells at the 6 h time point. Lower deuterium enrichment in downstream glucose metabolites is concomitantly supported by O_2_ consumption (Figure 5). Consistent with phenotypic glucose metabolism and β-lapachone drug action, treated NQO1^+^ cells consumed significantly lower O_2_ than control cells at the end of the [^2^H_7_]glucose tracer experiment (total time 8 h) whereas NQO1^-^ HCT-116 cells showed insignificantly lower O_2_ consumption in treated cells (Figure 5C,F,I). Previous work measured excess O_2_ consumption during the treatment period, but did not measure O_2_ consumption in a post-treatment setting [24]. This work indicates that a significant decrease in TCA cycle turnover is a durable effect of NQO1 bioactivation in the post-treatment phase.

To confirm the metabolic effects of β-lapachone treatment, a comparative analysis of 18 metabolites between control and treated cells in media was performed by GC-MS. The detected lactate level was significantly lower in β-lapachone treated cancer cells (Figure 6), consistent with interruption of glycolysis due to β-lapachone treatment [11]. Additionally, the metabolites panel showed disruptions in the production and consumption of key metabolites mediated by β-lapachone treatment with significant differences observed in NQO1^+^ cell lines and non-significant trends seen in NQO1^−^ HCT-116 cells. The lactate/alanine ratio reflects the NAD^+^/NADH equilibrium in the cytosol [44]. A549 cells have a control to treatment lactate/alanine ratio of ~7 while HCT-116 cells have a ratio of ~2; indicating a loss of reducing equivalents in A549 (NQO1^+^) β-lapachone treated cells. The intracellular metabolic panel also indicated that β-lapachone treatment perturbs the TCA cycle and amino acid metabolism, including branched-chain amino acids (BCAA) (Figure 6). As a whole, extracellular and intracellular metabolomic analysis alludes to potential avenues for β-lapachone combinatorial treatments targeting additional NAD^+^/NADH-dependent reactions. Significantly higher levels of intracellular BCAAs (valine, isoleucine, and leucine) in control NQO1^+^ cells (Figure 6), in contrast to extracellular levels (Figure S4), indicates that there is a possibility that β-lapachone action is inhibiting BCAA consumption by limiting NAD^+^/NADH availability of branched chained α-ketoacid dehydrogenase (BCKDH); a primary mediator of BCAA catabolism [45]. Similarly, significant differential levels of intracellular malate, aspartate, and asparagine observed between control and treated NQO1^+^ cells in contrast to non-significant extracellular differences suggest inhibition of the NADH dependent malate-aspartate shuttle [46]. BCAA catabolism or the malate-aspartate shuttle are logical targets for possible synergies with β-lapachone treatment.

## 5. Conclusions

Here, we report that HDO and ^2^H-lactate production can be used as a sensitive marker to measure differences in glycolytic metabolism caused by β-lapachone drug action. Recent results using [6,6-^2^H_2_]glucose as a tracer indicated that HDO production did not correlate well with glycolytic activity in an in vivo cancer model [47]. However, the amplified HDO production associated with the perdeuterated glucose might be sufficient to overcome the HDO signal associated with peripheral metabolism to yield an imaging biomarker for β-lapachone-mediated interruption of glycolysis. In vivo investigations of this hypothesis are underway. Deuterated lactate is an alternative biomarker for further investigation. It should be noted that the perdeuterated glucose tracer used here should produce more deuterated lactate, as both the C6 and C1 positions of glucose will label lacate-C3. Either of these markers could be used as inputs for an adaptive dosing model to reduce β-lapachone-associated toxicity that limits its use in human cancers at this time.

## Figures and Tables

**Table 1:** ^2^H mass balance between consumed [^2^H_7_]glucose and produced HDO and ^2^H-lactate.

Cell Lines		Control		Treatment	
A549		**Concentration (µmol/L)**	**Number of µmol of ^2^H**	**Concentration (µmol/L)**	**Number of µmol of ^2^H**
	[^2^H_7_]glucose	5064.0 ± 70.8	35,448.2 ± 496.1	1289.17 ± 148.67	9024.2 ± 1040.7
	HDO	19,623.3 ± 102.9	19,623.3 ± 552.2	6532.5 ± 851.6	6532.5 ± 851.6
	Lactate-CH_2_D	4663.4 ± 136.2	4663.4 ± 136.2	1279.3 ± 147.6	1279.3 ± 147.6
	Lactate-CHD_2_	3034.0 ± 105.3	6068.0 ± 210.6	595.3 ± 83.6	1190.5 ± 167.2
	µmol of ^2^H in ^2^H-lactate and HDO		30,354.8		9002.3
MiaPaCa2	[^2^H_7_]glucose	5723.0 ± 39.4	40,061.1 ± 276.4	2199.6 ± 21.7	15,397.2 ± 152.3
	HDO	22,344.5 ± 471.6	22,344.5 ± 471.6	10,629.0 ± 526.4	10,629.0 ± 526.4
	Lactate-CH_2_D	4798.8 ± 122.4	4798.8 ± 122.4	1562.1 ± 116.5	1562.1 ± 116.5
	Lactate-CHD_2_	3046.3 ± 68.0	6092.6 ± 135.9	753.8 ± 59.6	1507.6 ± 119.3
	µmol of ^2^H in ^2^H-lactate and HDO		33,075.9		13,098.8
HCT-116	[^2^H_7_]glucose	4373.3 ± 19.2	30,613.7 ± 427.1	3510.6 ± 48.4	24,574.6 ± 338.9
	HDO	13,004.6 ± 494.6	13,004.6 ± 494.6	10,854.5 ± 786.38	10,854.5 ± 786.3
	Lactate-CH_2_D	3151.1 ± 242.4	3151.1 ± 242.4	2607.2 ± 149.9	2607.2 ± 149.9
	Lactate-CHD_2_	2397.7 ± 229.5	4795.5 ± 459.0	1967.1 ± 145.9	3934.1 ± 291.9
	µmol of ^2^H in ^2^H-lactate and HDO		20,951.2		17,395.8

^2^H mass balance between µmols of ^2^H atoms of consumed [^2^H_7_]glucose and µmols of ^2^H atoms incorporated into effluxed ^2^H-lactate and HDO by control and treated A549, MiaPaCa2 and HCT-116 cancer cells at 6 h post-treatment time points. (Note: [^2^H_7_]glucose, ^2^H-lactate and HDO were measured from ^2^H-NMR data represented as mean ± SEM).

## Data Availability

The data presented in this study are available in this article and Supplementary Materials and will also be available from the corresponding author upon request.

## Supplementary Materials

The following are available online at https://www.mdpi.com/article/10.3390/cancers13164165/s1, Figure S1: Comparative analysis shows reduced glycolytic flux in NQO1^+^ β-lapachone treated cells, Figure S2: Kinetic analysis of glycolysis in MiaPaCa2 cells, Figure S3: Time course measurement of glycolytic rate in HCT-116 cells, Figure S4: Extracellular metabolic panel shows differential level of metabolites between control and treated A549 cells, Figure S5: Extracellular differential level of metabolites in MiaPaCa2 cells, Figure S6: Analysis of the extracellular metabolites in HCT-116 cells, Figure S7: Intracellular levels of metabolites in HCT-116 cells, Figure S8: Schematic diagram representing the experimental timeline, Figure S9: The ^2^H-labeling in the metabolites of control and treated cells, Table S1: Kinetic analysis during β-lapachone treatment, Table S2: Kinetic analysis data of post-treatment periods.
