# Supplementary material for: Measuring NQO1 Bioactivation Using [2H7]Glucose
Source: Cancers (Basel). 2021 Aug 19;13(16):4165. doi: 10.3390/cancers13164165 (PMC8392257; doi:10.3390/cancers13164165)
Supplement: Supplementary file 1 [file cancers-13-04165-s001.zip › cancers-1292790-supplementary.pdf]

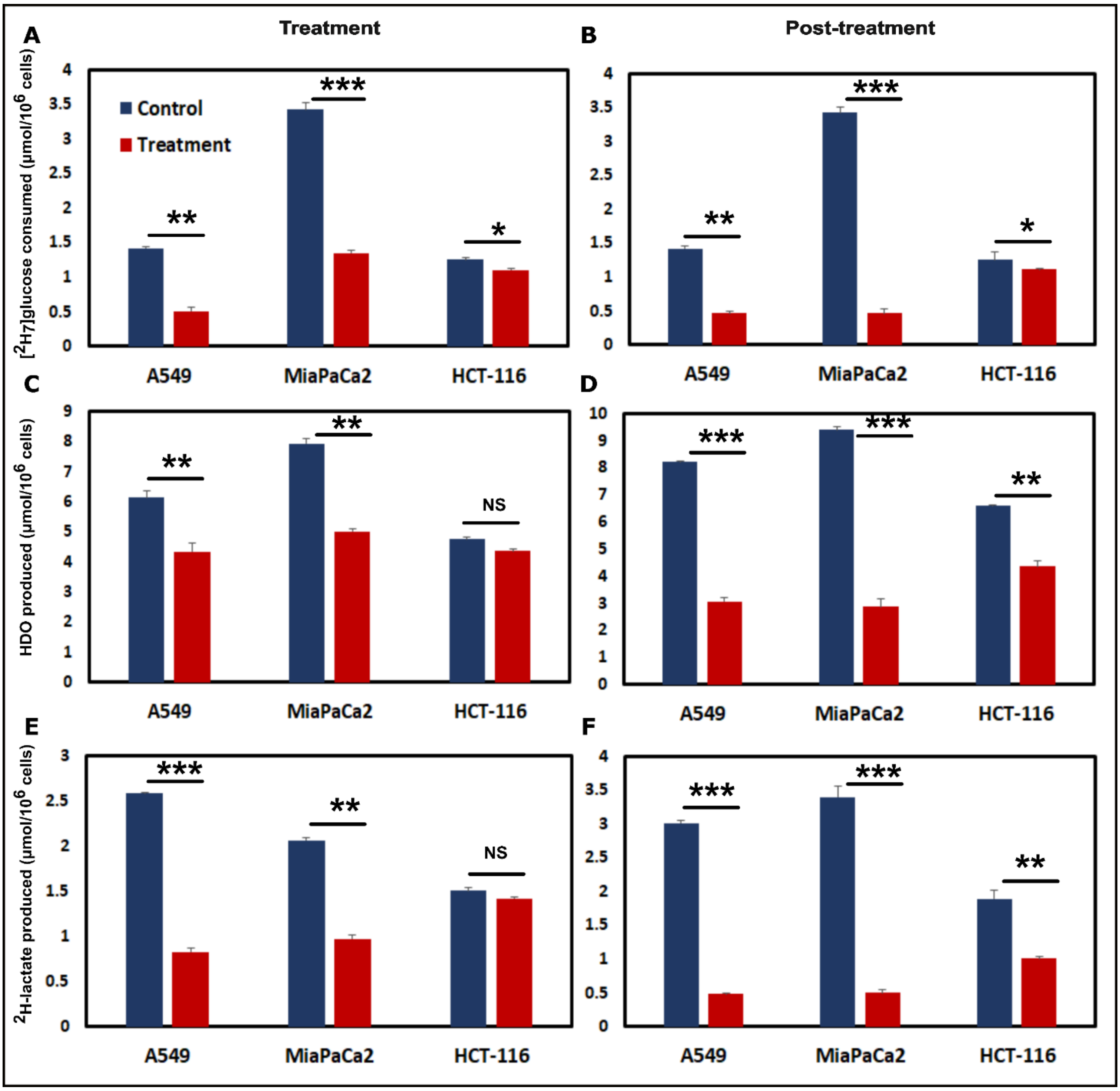

Figure S1. Comparative analysis shows reduced glycolytic flux in NQO1+ β-lapachone treated cells. Bar plots of [$^2H_7$]glucose consumption, HDO and ^{2}H-lactate production by A549, MiaPaCa2 and HCT-116 cells during treatment (120 min time point, Panels, **A, C** and **E**) and post-treatment (Panels, **B, D** and **F**) periods at 120 min time point (total time 240 minutes). HDO and ^{2}H-lactate production were significantly higher for control A549 and MiaPaCa2 cells compared to treatment counterparts whereas it was insignificant for HCT-116 cells during treatment but significant for the post-treatment period. (Note: 120 min time points were chosen for all of the three cancer cell lines to compare cellular [$^2H_7$]glucose uptake and HDO and ^{2}H-lactate production during the treatment and post-treatment periods. *N*=3: biological replicate data is represented as mean ± standard error of mean (SEM). Significant level was calculated using the Student's t-test between control and treated groups and has been displayed as: non-significant 'NS' if P>0.05, '*' if P≤0.05, '**' if P≤0.01,'***' if P≤0.001).

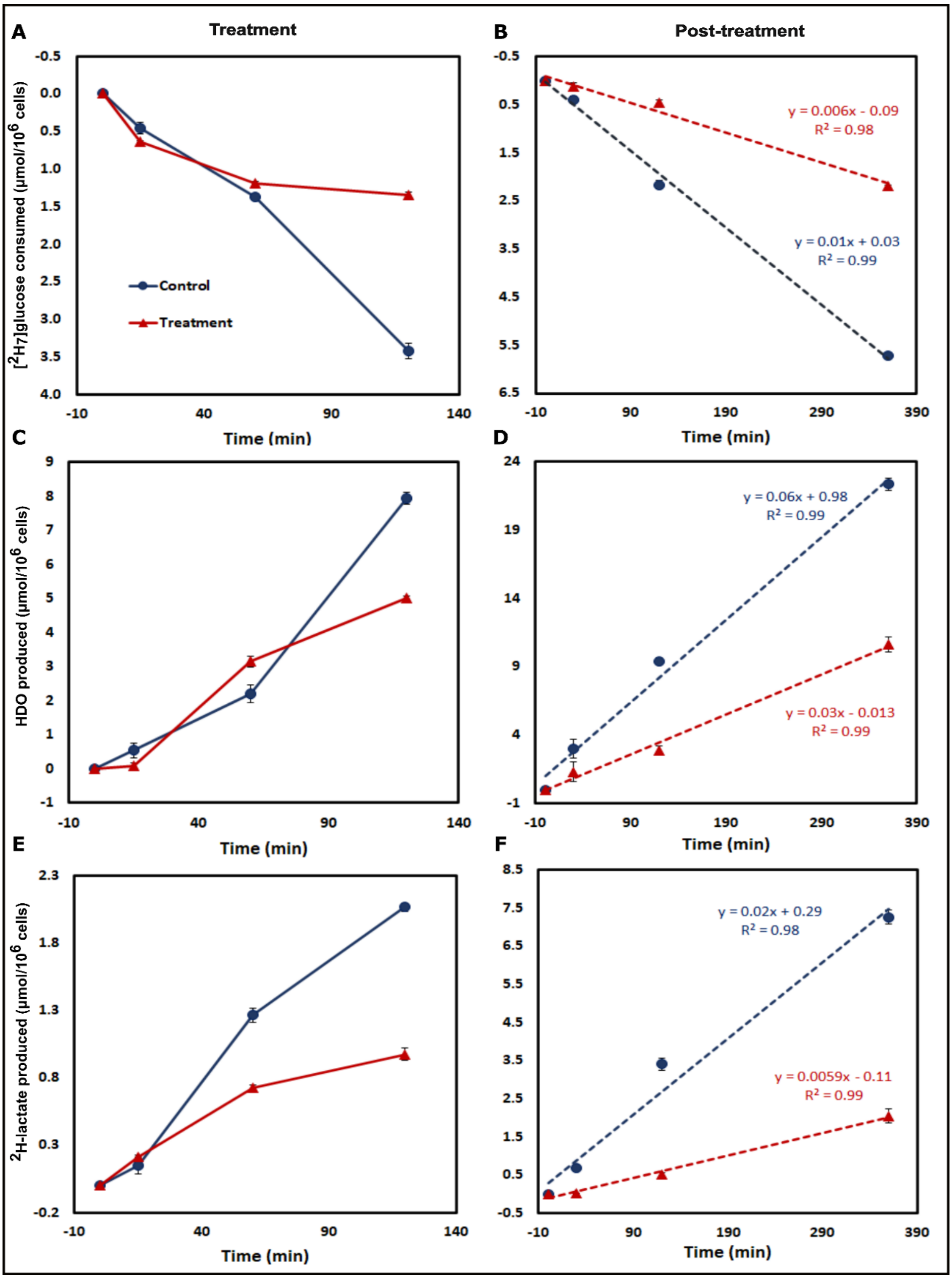

Figure S2. Kinetic analysis of glycolysis in MiaPaCa2 cells. Time series plots of [$^2H_7$]glucose consumption (Panels, **A** and **B**), HDO (Panels, **C** and **D**) and ^{2}H-lactate production (Panels, **E** and **F**) by control and treated MiaPaCa2 cells during treatment (left column) and post-treatment (right column) periods. (Note: Residual [$^2H_7$]glucose, HDO and ^{2}H-lactate concentrations were measured by ^{2}H-NMR in the cell media samples withdrawn at 0 min, 15 min, 1 h, and 2 h (treatment) and 0 min, 30 min, 2 h, and 6 h (post-treatment) time points of experiment. *N*=3: biological replicate data is represented as mean ± SEM).

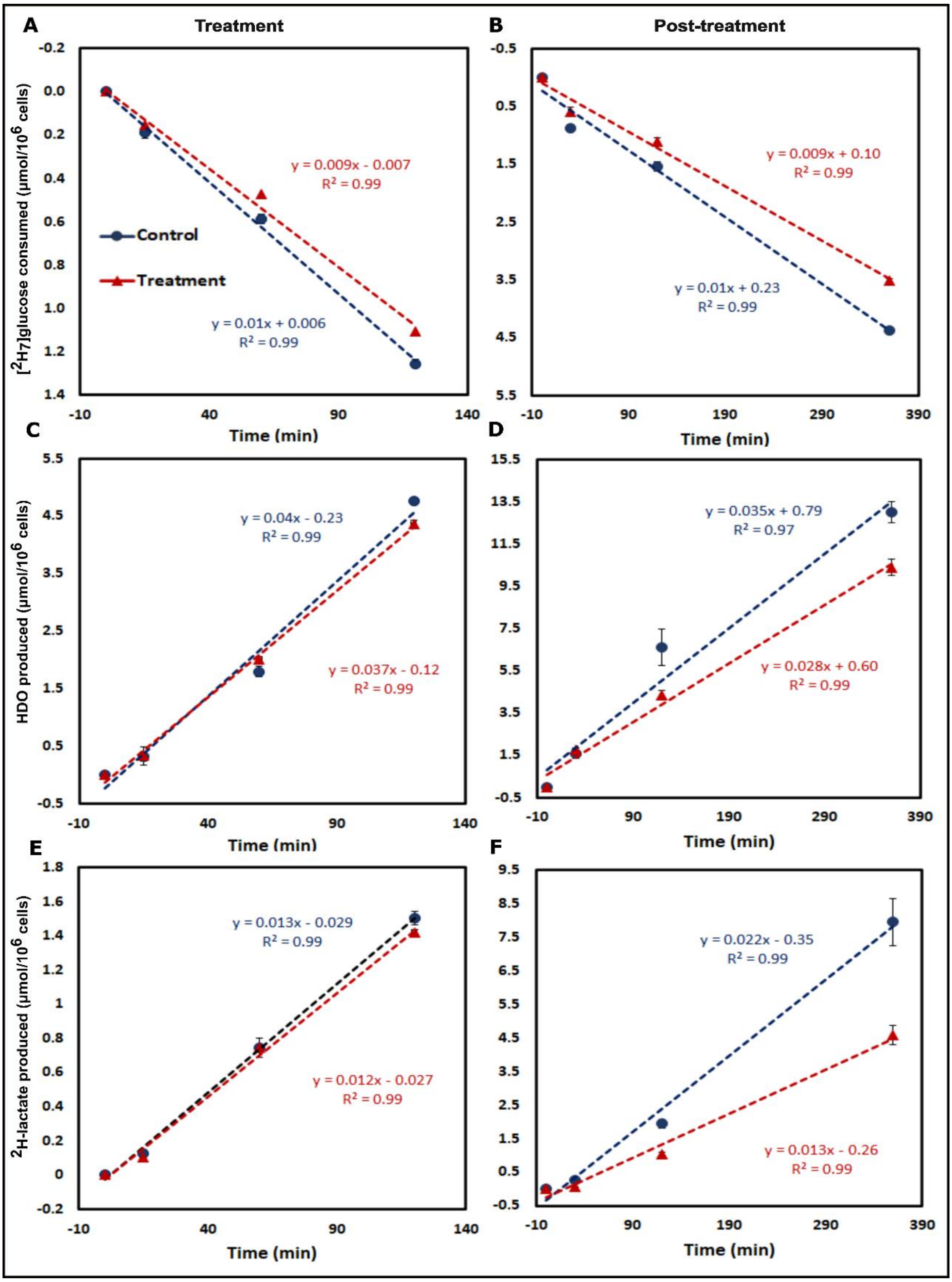

Figure S3. Time course measurement of glycolytic rate in HCT-116 cells. Kinetic plots of [$^2H_7$]glucose consumption (Panels, **A** and **B**), HDO (Panels, **C** and **D**) and ^{2}H-lactate production (Panels, **E** and **F**) by control and treated HCT-116 cells during treatment (left column) and post-treatment (right column) periods. (Note: Residual [$^2H_7$]glucose, HDO and ^{2}H-lactate concentrations were measured by ^{2}H-NMR in the cell media samples withdrawn at 0 min, 15 min, 1 h, and 2 h (treatment) and 0 min, 30 min, 2 h, and 6 h (post-treatment) time points. *N*=3: biological replicate data is represented as mean ± SEM).

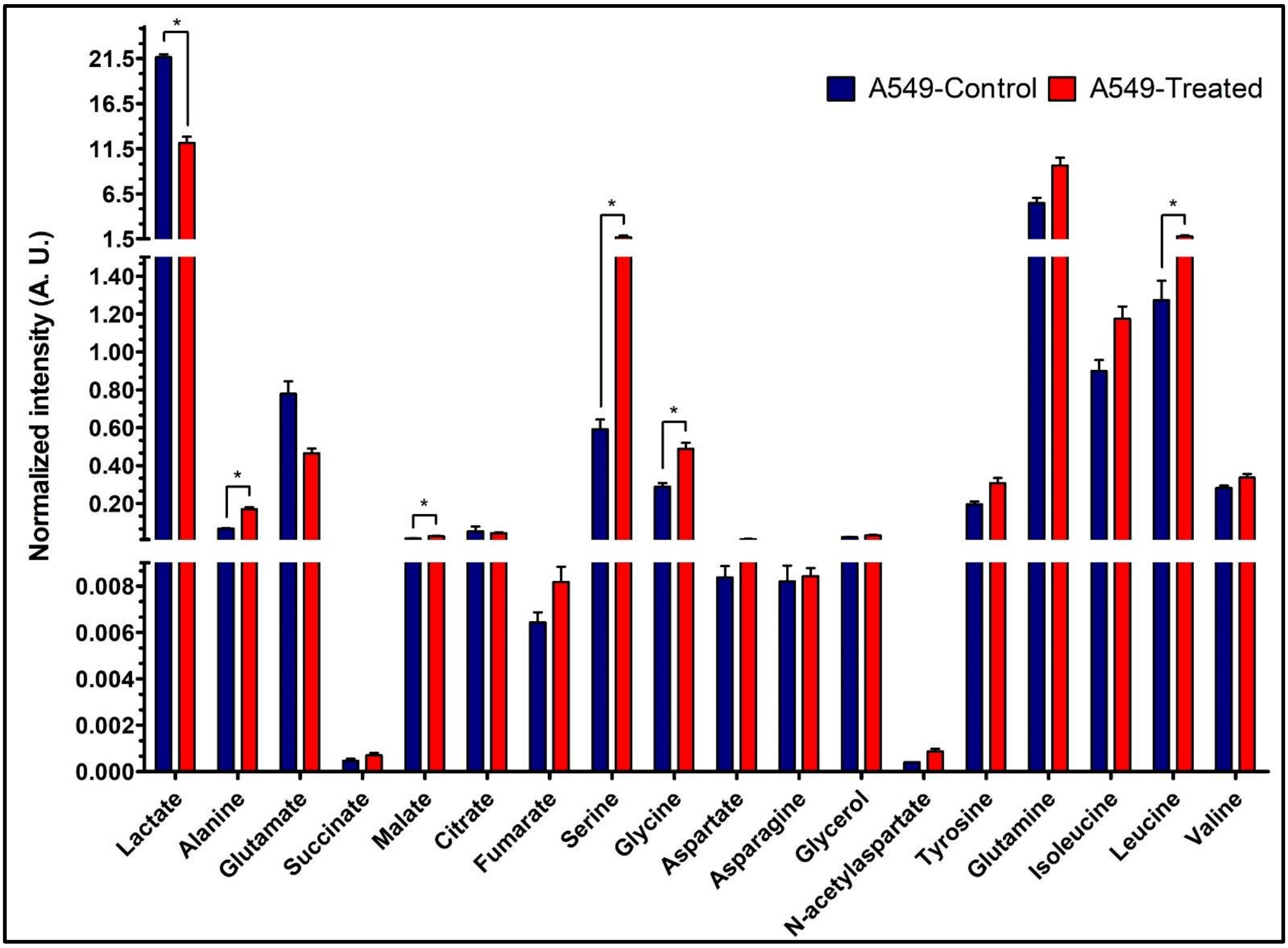

Figure S4. Extracellular metabolic panel shows differential level of metabolites between control and treated A549 cells. GC-MS analysis of 18 metabolites shows differential levels between control and treated A549 cells. The cell media of treated A549 cells showed significantly higher production levels of alanine and malate. In contrast, higher levels of lactate were detected in control cell media suggesting that β-lapachone treatment impairs lactate production. Analysis of cellular consumption showed significantly higher levels of serine, glycine, and leucine in the cell media of treated cells. (Note: *N*=3: biological replicate data is represented as mean ± SEM. Statistical significance was determined by employing the Student's t-test followed by false discovery rate (FDR) approach to adjust P values and has been represented as: '*' if P≤0.05).

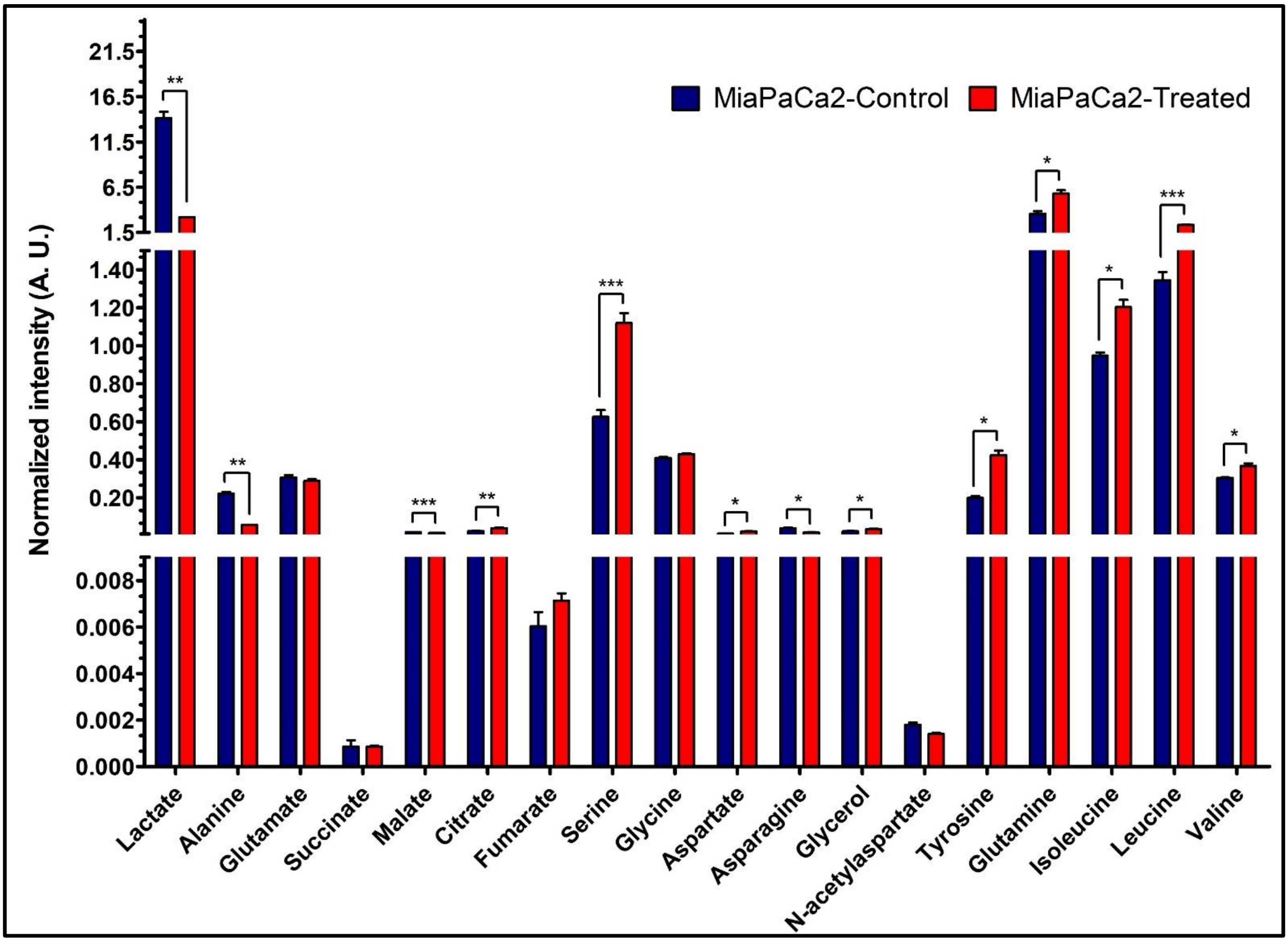

Figure S5. Extracellular differential level of metabolites in MiaPaCa2 cells. Bar plots of GC-MS detected levels of 18 different extracellular metabolites from MiaPaCa2 cells. Representative graphs of production and consumption analysis show a comparison between control and treated cells. Cell media samples of treated MiaPaCa2 cells show significantly lower production of lactate, alanine, malate, and asparagine, while showing significantly higher production of citrate, aspartate, and glycerol. Extracellular metabolite consumption analysis shows that compared to control cells, treated cells consumed significantly lower levels of serine, tyrosine, glutamine, isoleucine, leucine, and valine. (Note: *N*=3: biological replicate data is represented as mean ± SEM. Student's t-test was used to calculate statistical significance followed by false discovery rate (FDR) approach to adjust P values and has been represented as: '*' if P≤0.05, '**' if P≤0.01,'***' if P≤0.001).

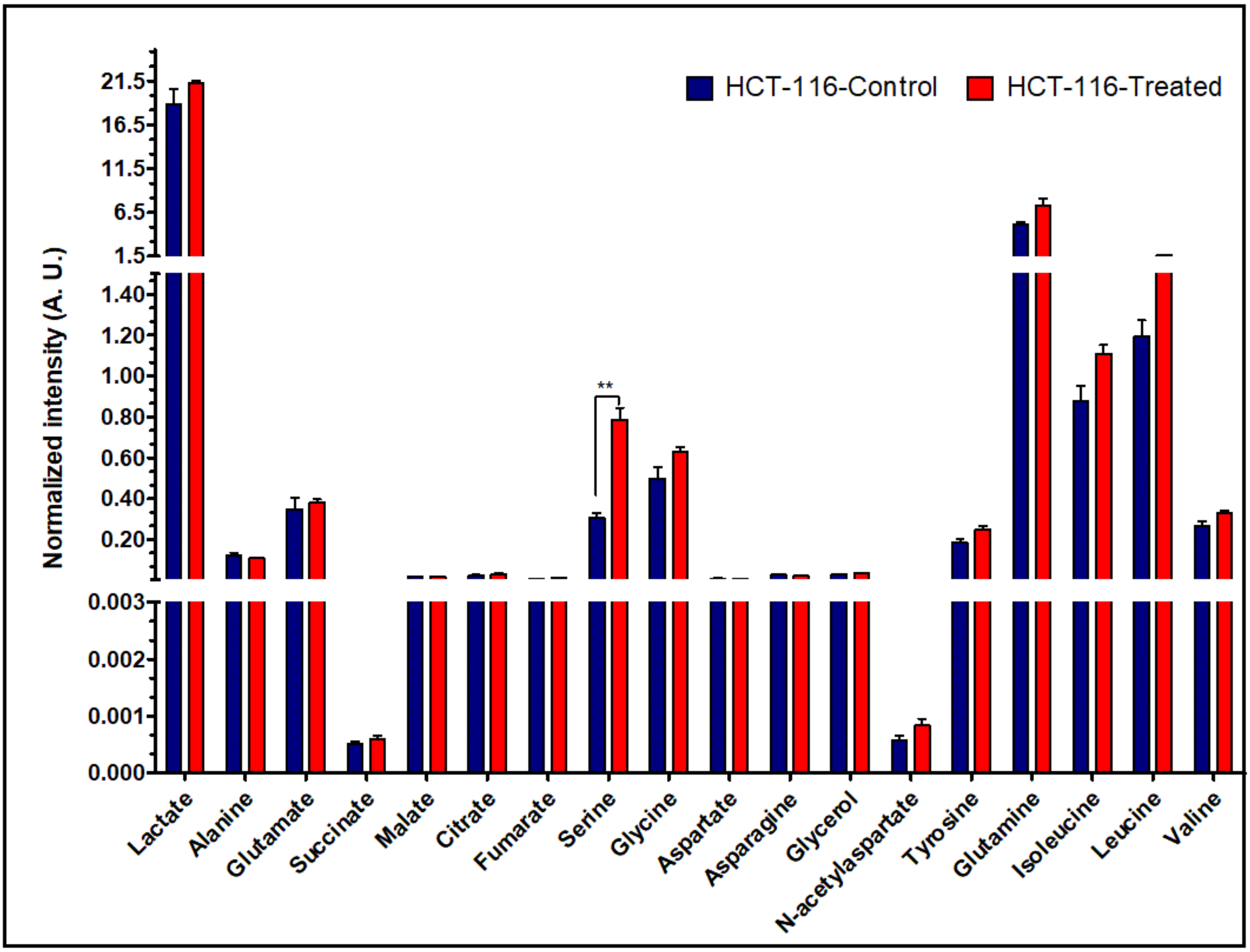

Figure S6. Analysis of the extracellular metabolites in HCT-116 cells. Production and consumption of extracellular metabolite analysis of control and treated HCT-116 cells. Bar plots were assembled from mean ± SEM data from biological triplicates. Treatment cell media showed a production trend of higher levels of lactate, glutamate, succinate, citrate, fumarate, glycerol, and N-acetylaspartate. Consumption of serine, glycine, tyrosine, isoleucine, leucine, and valine was lower in treatment cells. (Note: *N*=3: biological replicate data is represented as mean ± SEM. Student's t-test was used to calculate statistical significance followed by false discovery rate (FDR) approach to adjust P values and has been represented as: '**' if P≤0.01).

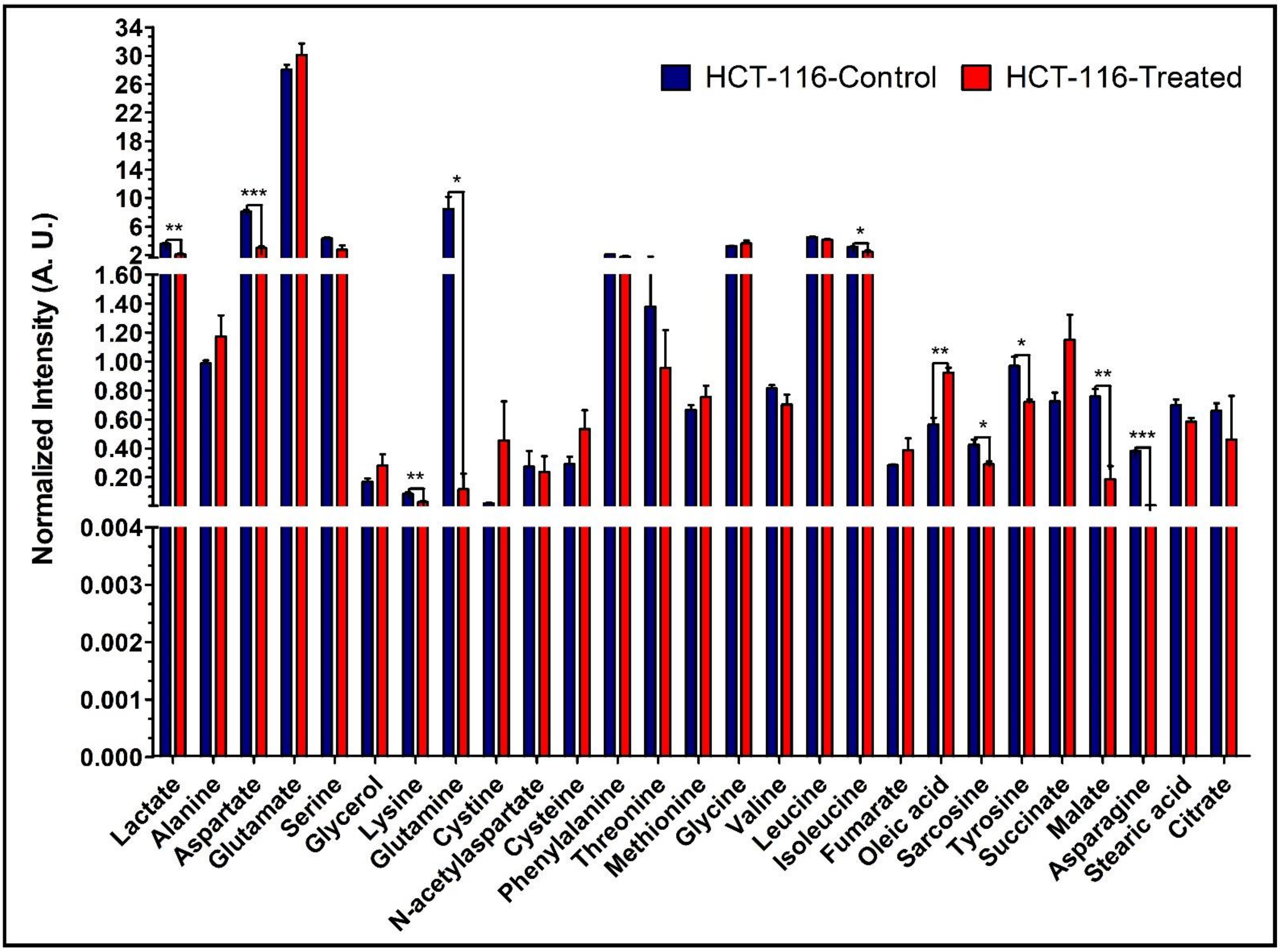

Figure S7. Intracellular levels of metabolites in HCT-116 cells. Bar plots display the differential normalized signal intensity of the metabolites between control and treated HCT-116 cells. (Note: *N*=3: biological replicate data is represented as mean ± SEM. Student's t-test was used to calculate statistical significance followed by false discovery rate (FDR) approach to adjust P values and represented as: '*' if P≤0.05, '**' if P≤0.01, '***' if P≤0.001).

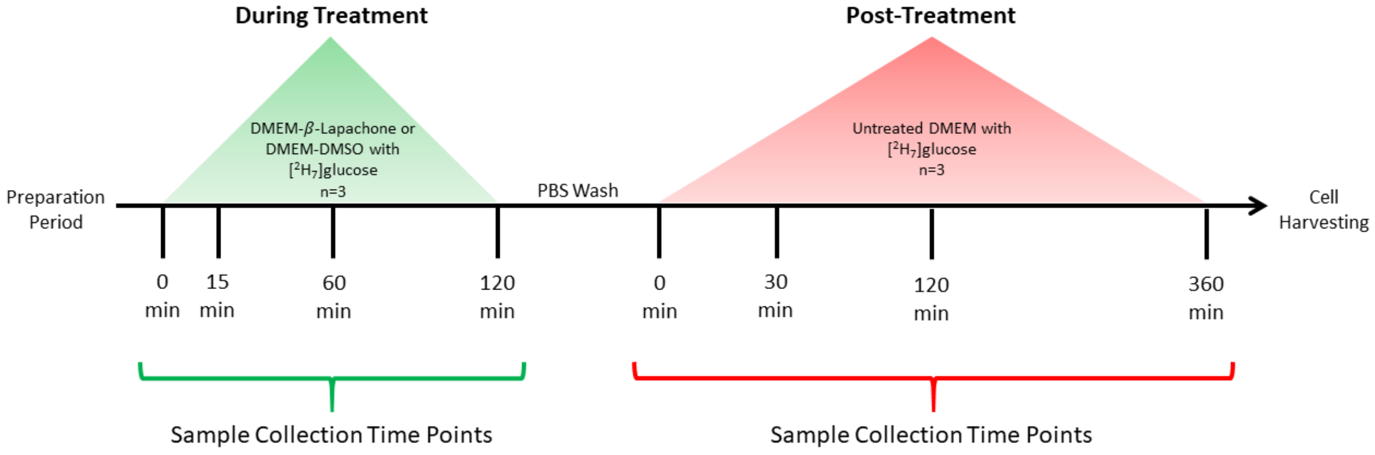

Figure S8. Schematic diagram representing the experimental timeline. Preparation period represents adding treatment media to cells to start the experiment. During treatment period took place over 2 hours (120 min). At the end of the treatment period, media was removed and cells were washed with PBS to remove any residual -lapachone and DMSO. Immediately after, untreated DMEM with [$^2H_7$] glucose was added to the cells to start the post-treatment period. Finally, cells were harvested after 6 hours (360 min) of post-treatment period.

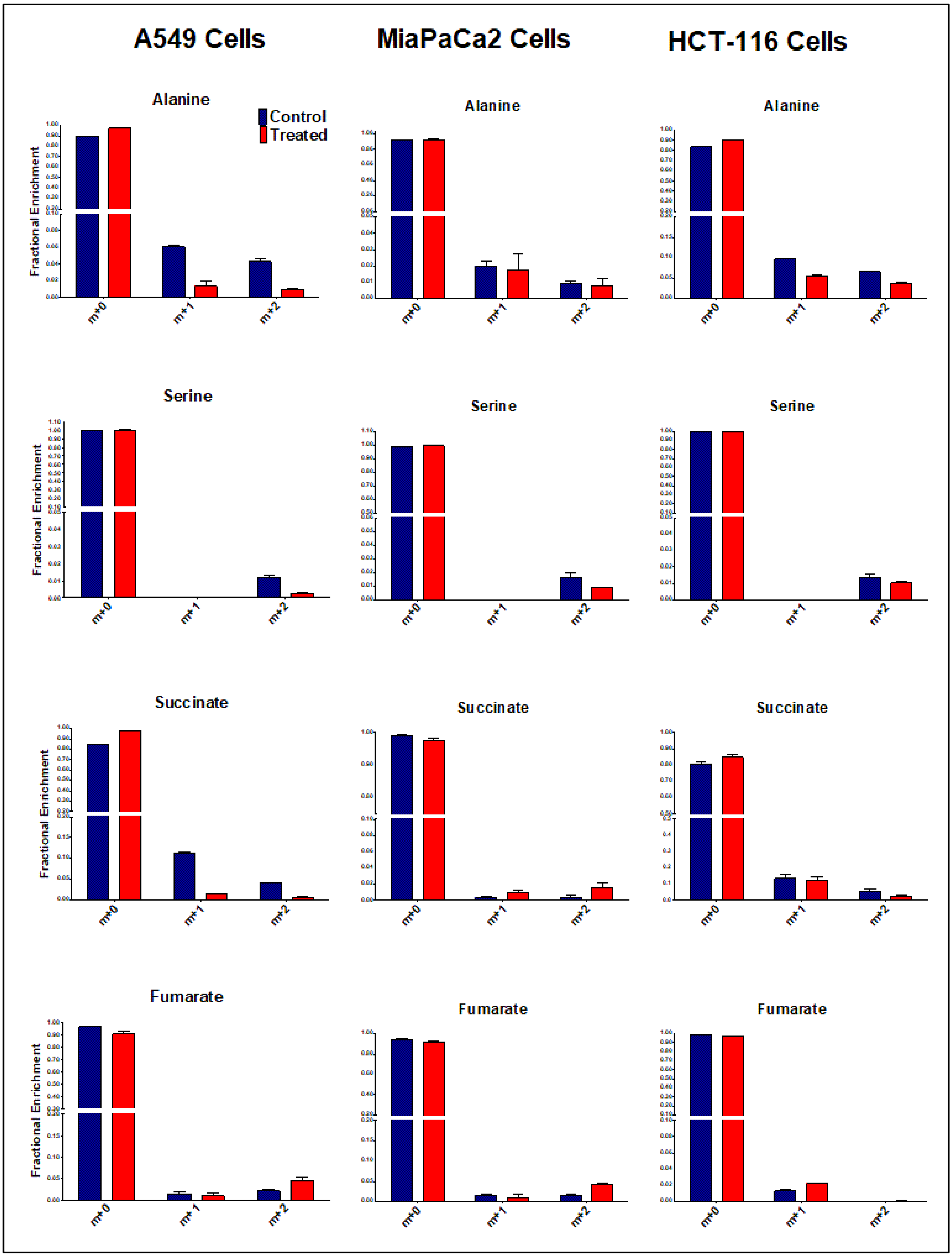

Figure S9. The ^{2}H-labeling in the metabolites of control and treated cells. Enrichment results were calculated from the GC-MS data of samples collected at 360 min post-treatment time point. The GC-MS data was corrected for natural isotope abundance using the Isotopomer Network Compartmental Analysis (INCA) software. Fractional enrichments for m+1 and m+2 isotopologues represent the ^{2}H deposition from [$^2H_7$]glucose metabolism in control and treated cells.

Table S1. Kinetic analysis during β-lapachone treatment.

Time series data during β-lapachone treatment period						
	**Glucose (μmol/10⁶ cells)**		**HDO (μmol/10⁶ cells)**		**Lactate (μmol/10⁶ cells)**	
**Time points**	**Control**	**Treatment**	**Control**	**Treatment**	**Control**	**Treatment**
**A549 Cells**						
**15 min**	0.45 ± 0.03	0.31 ± 0.08	1.02 ± 0.81	0.51 ± 0.25	0.05 ± 0.03	0.05 ± 0.03
**60 min**	0.70 ± 0.09	0.40 ± 0.01	2.88 ± 0.17	2.78 ± 0.15	0.98 ± 0.07	0.48 ± 0.03
**120 min**	1.42 ± 0.03	0.51 ± 0.06	6.12 ± 0.23	4.33 ± 0.29	2.59 ± 0.01	0.83 ± 0.04
**Slope (μmol/min)**	NA	NA	*0.050± 0.005	0.037± 0.003	***0.022± 0.0006	0.007± 0.004
**MiaPaCa2 Cells**						
**15 min**	0.46 ± 0.08	0.64 ± 0.01	0.54 ± 0.21	0.07 ± 0.10	0.15 ± 0.06	0.21 ± 0.02
**60 min**	1.38 ± 0.03	1.19 ± 0.02	2.20 ± 0.26	3.15 ± 0.15	1.26 ± 0.05	0.73 ± 0.02
**120 min**	3.43 ± 0.10	1.35 ± 0.04	7.93 ± 0.18	5.01 ± 0.07	2.07 ± 0.03	0.97 ± 0.04
**Slope (μmol/min)**	NA	NA	NA	NA	NA	NA
**HCT-116 Cells**						
**15 min**	0.19 ± 0.03	0.16 ± 0.03	0.33 ± 0.08	0.34 ± 0.16	0.13 ± 0.02	0.10 ± 0.01
**60 min**	0.59 ± 0.02	0.47 ± 0.02	1.80 ± 0.09	2.01 ± 0.05	0.74 ± 0.05	0.75 ± 0.01
**120 min**	1.26 ± 0.02	1.11 ± 0.02	4.76 ± 0.06	4.36 ± 0.07	1.50 ± 0.04	1.42 ± 0.02
**Slope (μmol/min)**	**0.01 ± 0.0001	0.009 ±0.0003	0.040 ± 0.001	0.037 ± 0.0006	0.013 ± 0.0002	0.012 ± 0.001

The concentration of [$^2H_7$]glucose, HDO and ^{2}H-lactate in control and β-lapachone treated A549, MiaPaCa2 and HCT-116 cancer cells at 15, 60, and 120 min time points for the treatment period. The calculated slopes from the time series data are also shown for control and treated cells. *N*=3: biological replicate data is represented as mean ± SEM. Significance levels between control and treatment groups were calculated using the Student's t-test in the Graphpad prism and shown as: as: '*' if P≤0.05, '**' if P≤0.01, '***' if P≤0.001. NA is not available due to the non-linear line for time series data.

Table S2. Kinetic analysis data of post-treatment periods.

Time series data of the post-treatment period						
	**Glucose (μmol/10⁶ cells)**		**HDO (μmol/10⁶ cells)**		**Lactate (μmol/10⁶ cells)**	
**Time points**	**Control**	**Treatment**	**Control**	**Treatment**	**Control**	**Treatment**
**A549 cells**						
**30 min**	0.22 ± 0.16	0.25 ± 0.24	3.02 ± 0.68	1.29 ± 0.73	0.56 ± 0.07	0.08 ± 0.03
**120 min**	1.78 ± 0.04	0.46 ± 0.04	8.22 ± 0.24	3.06 ± 0.16	3.24 ± 0.04	0.53 ± 0.01
**360 min**	5.06 ± 0.07	1.29 ± 0.15	19.62 ± 0.55	6.53 ± 0.85	7.70 ± 0.23	1.87 ± 0.23
**Slope (μmol/min)**	***0.014 ± 0.0004	0.003 ± 0.0006	***0.053 ± 0.002	0.017 ± 0.003	***0.021 ± 0.001	0.005 ±0.001
**MiaPaCa2 Cells**						
**30 min**	0.40 ± 0.07	0.13 ± 0.08	2.19 ± 0.43	1.93 ± 0.87	0.73 ± 0.05	0.04 ± 0.01
**120 min**	2.17 ± 0.08	0.46 ± 0.06	9.40 ± 0.25	2.86 ± 0.31	3.66 ± 0.17	0.56 ± 0.05
**360 min**	5.72 ± 0.04	2.20 ± 0.02	22.34 ± 0.47	10.63 ± 0.53	7.85 ± 0.18	2.32 ± 0.18
**Slope (μmol/min)**	***0.016 ± 0.0002	0.006 ± 0.0007	***0.06 ± 0.002	0.03 ± 0.0005	***0.02 ± 0.0004	0.007 ± 0.0005
**HCT-116 Cells**						
**30 min**	0.89 ± 0.04	0.59 ± 0.03	1.59 ± 0.26	1.75 ± 0.07	0.25 ± 0.04	0.05 ± 0.02
**120 min**	1.54 ± 0.11	1.11 ± 0.01	6.60 ± 0.87	4.35 ± 0.19	1.94 ± 0.13	1.04 ± 0.04
**360 min**	4.37 ± 0.06	3.55 ± 0.03	13.00 ± 0.49	10.37 ± 0.38	7.95 ± 0.70	4.57 ± 0.29
**Slope (μmol/min)**	**0.012 ± 0.0001	0.009 ± 0.0004	*0.035 ± 0.001	0.028 ± 0.001	*0.016 ± 0.002	0.013 ± 0.001

The concentration of [$^2H_7$]glucose, HDO and ^{2}H-lactate in control and β-lapachone treated A549, MiaPaCa2 and HCT-116 cancer cells at 30, 120 and 360 min time points for the post-treatment period. The slopes from the time series data are also shown for control and treated cells. Biological triplicate data is represented as mean ± standard error of mean (SEM). Significance levels between control and treatment groups were calculated using the Student's t-test in the Graphpad prism and represented as: '*' if P≤0.05, '**' if P≤0.01, '***' if P≤0.001.
